# Effects of access to primary healthcare services on health vulnerability among older adults in China: focusing on demographic differences

**DOI:** 10.3389/fpubh.2025.1656886

**Published:** 2025-10-13

**Authors:** Ningli Zhu, Jun Li, Xiaohui Liu, Lei Luo, Wenwen Cheng, Xiaoyu Li, Chen Yu, Siyu Zhang, Liang Zhu

**Affiliations:** ^1^Department of Health Service Management and Medical Education, School of Preventive Health, The Fourth Military Medical University, Xi'an, Shaanxi, China; ^2^The Ministry of Education Key Lab of Hazard Assessment and Control in Special Operational Environment, Xi'an, Shaanxi, China; ^3^The Shaanxi Provincial Key Laboratory of Environmental Health Hazard Assessment and Protection, Xi'an, Shaanxi, China; ^4^The First Affiliated Hospital of the Fourth Military Medical University, Xi'an, Shaanxi, China; ^5^Xijing 986 Hospital Department, The Fourth Military Medical University, Xi'an, Shaanxi, China

**Keywords:** ageing, primary healthcare services, health vulnerability, structural equation modeling, comparative analysis

## Abstract

**Background:**

Against the backdrop of an aging population, older adults are confronted with multifaceted challenges, including physiological decline, uneven distribution of medical resources, and economic constraints, which collectively exacerbate their health vulnerability. This study aims to examine the impact of primary healthcare accessibility on health vulnerability among older adults and to explore disparities in access to primary care services across different demographic groups, thereby providing an empirical foundation for promoting healthy aging.

**Methods:**

An online survey was conducted among 398 older adults in China to assess health vulnerability and accessibility to village clinics and township health centers. A baseline model and a multi-group structural equation model were used to analyze the effect of primary healthcare accessibility on health vulnerability.

**Results:**

While overall health vulnerability among older adults remained relatively high, accessibility to primary healthcare services was generally favorable. Compared to village clinics, township health centers have significantly higher accessibility. Furthermore, demographic variables such as gender, education level, and marital status demonstrated heterogeneous effects within this relationship.

**Conclusion:**

Policy efforts should prioritize enhancing resource allocation and service capacity at the village clinic level. Concurrently, greater attention should be devoted to ensuring healthcare equity for vulnerable subgroups, including women, those living alone, and individuals with lower educational attainment, within the primary healthcare system. These measures will contribute to reducing health vulnerability among older adults and advancing the goal of healthy aging.

## 1 Introduction

Population aging is accelerating globally. According to the United Nations, the population aged 65 and above is projected to exceed that under the age of 18 by the end of this century (1). This demographic shift not only transforms population structures but also imposes substantial pressures on healthcare systems worldwide (2). As the proportion of older adults rises, age-related physiological decline increases their susceptibility to chronic diseases, falls, and functional impairments. Furthermore, older adults face compounded challenges stemming from the inevitability of biological aging, inequitable distribution of medical resources, and financial constraints. Together, these factors exacerbate health vulnerabilities among older adults, presenting complex new challenges for health policymakers and providers.

Globally, primary healthcare serves as the foundational “gatekeeper” and “coordinator” within health systems for older adults (3). In China, as a critical component of the national health system, primary healthcare (PHC) acts as a key entry point for individuals, families, and communities—particularly at the township level—ensuring equitable access to essential health services (4). It provides preventive, therapeutic, and rehabilitative care tailored to the needs of the aging population (5). Furthermore, PHC is integral to the hierarchical medical system (6), which coordinates medical resources to enhance service delivery at county and township health centers, especially in underserved regions (7). Nevertheless, the primary healthcare system continues to face significant challenges, including shortages of human resources, insufficient infrastructure, fragile supply chains, and persistent inequities in service accessibility across different geographic areas (8).

Health vulnerability represents a multidimensional and integrative concept encompassing biological, psychological, and social dimensions, allowing for a more comprehensive assessment of health status among older adults (9). The vulnerability of aging populations is attracting increasing scholarly attention. Existing literature reflects divergent perspectives on the relationship between “vulnerability” and “frailty” in older adults: some scholars equate the two, conceptualizing frailty as a manifestation of vulnerability that intensifies with age; others contend that the concepts are distinct, with vulnerability potentially leading to frailty and ultimately contributing to declines in both physical and cognitive function (10). Aligning with the first perspective, this study examines the impact of primary healthcare accessibility on health vulnerability among older adults. We define health vulnerability in this population as follows: due to specific physiological, psychological, or socioeconomic conditions, older adults experience heightened health threats that increase their risk of illness or diminish their capacity to cope with health challenges. This condition is influenced by both individual and external factors. Individual factors include age, gender, education, income, and lifestyle, while external factors encompass environmental conditions, educational resources, income distribution, employment opportunities, social support, and healthcare services (11, 12). Regarding the measurement of health vulnerability, this study uses the Frailty Index (FI) developed by Rockwood, a widely adopted instrument for assessing health deficits in older adults (13). The FI integrates multiple health deficits into a composite index, overcoming the limitations of single-dimension assessments and improving comparability across populations (14). It incorporates two assessment paradigms: the physiological phenotype model, which emphasizes biomarkers and functional decline, and the cumulative deficit model, which quantifies health risks by calculating the ratio of accumulated health deficits to the total number of health indicators considered (13, 15, 16).

Although existing studies vary in their selection of indicators for health vulnerability among older adults, they consistently emphasize the interrelationship and underlying mechanisms between physical and mental health vulnerability. Data from 2020 show that primary healthcare institutions accounted for only 10% of the total healthcare service utilization nationwide (17). Notably, medical service utilization among older adults in rural China remains particularly low, with as many as 39.4% not seeking medical care within a 2-week period when needed (18). Primary healthcare services exert a substantial influence on health vulnerability in the older adult population. High-quality primary care, especially within the public health system, can effectively mitigate vulnerability resulting from diverse risk conditions, owing to its attributes of accessibility and continuity of care (19). Previous research has demonstrated that community health services and long-term care can significantly improve both objective and subjective health outcomes while reducing older adults’ vulnerability to poor health (20, 21). These services are typically delivered by primary healthcare workers within designated geographical areas.

In summary, researchers internationally have conducted extensive studies on health vulnerability among older adults, building upon a well-established theoretical and methodological foundation. However, conceptual disagreements persist regarding the precise definition and scope of health vulnerability in this population. Moreover, much of the existing literature focuses on macro-level assessments or uses multivariate regression to examine the status and determinants of health vulnerability. Although the relationship between healthcare services and health outcomes is well-established across populations, the majority of this evidence relies solely on self-reported health measures. Limited research has been conducted to specifically assess the impact of primary healthcare accessibility on health vulnerability. Therefore, this study aims to investigate how access to primary healthcare services—examined at two tiers of the Chinese healthcare system—affects health vulnerability among older adults, with particular emphasis on demographic variations.

This study aims to examine the impact of primary healthcare accessibility on health vulnerability among older adults, with a specific focus on the roles of village clinics and township health centers. It further investigates heterogeneity in this relationship across different social groups, thereby providing an evidence-based foundation for promoting healthy aging. The current research extends existing literature by examining the aforementioned associations through three primary approaches. First, we assess the current status of health vulnerability among older adults in China across both physiological and psychological health dimensions. Second, we develop a Structural Equation Model (SEM) to evaluate the effects of accessibility to primary healthcare services at both village clinics and township health centers on health vulnerability. Third, acknowledging potential variation in the effects of primary healthcare services, we use multi-group analysis to explore differential impacts across diverse demographic groups.

## 2 Methods

### 2.1 Sample and sampling

This survey was conducted among older adults permanently residing in rural areas of China during July and August 2024. Given the extensive geographic scope, along with practical constraints related to time, funding, and varying levels of digital literacy among the older adult population, the study used the Credamo online data platform for randomized questionnaire distribution. To overcome challenges associated with self-reporting, particularly among very old or socially isolated individuals, and to enhance sample representativeness while minimizing bias, a proxy-response method was adopted, whereby adult children provided responses on behalf of their parents. All participants were required to answer every item in the questionnaire.

To ensure the smooth implementation of the online survey, a two-phase survey approach was adopted. In the first phase, we conducted field visits to five villages, collected over 50 questionnaire responses, and held face-to-face discussions with multiple older adults and their children. Based on the feedback received, the questionnaire was refined and optimized through multiple rounds of revisions to ensure clear and comprehensible wording of the questions, to enhance its appropriateness for the target population, and to establish a solid foundation for the validity of proxy-completed questionnaires by adult children. The second phase involved the formal survey. Eligibility criteria included respondents aged 60 years or older, proxies possessing comprehensive knowledge of their parents’ health status, and long-term residence in rural areas. Sample size was determined using the Kendall method, which recommends 10 to 20 observations per variable (22). Accounting for an anticipated 10% attrition rate, the target sample size was set between 200 and 400 participants. To ensure data quality, several filtering mechanisms were implemented, including IP address verification and account restrictions to prevent duplicate entries. Additional checks excluded responses with completion times under 5 min or those exhibiting logical inconsistencies. From an initial pool of 501 collected questionnaires, 103 were excluded due to overly rapid completion, missing data, or failure to meet residency or age criteria, resulting in 398 valid responses for analysis. Statistical analysis confirmed that the sample was well-distributed geographically, supporting its randomness and indicating successful mitigation of selection bias.

### 2.2 Variable definitions and descriptions

#### 2.2.1 The dependent variable

In this study, the dependent variable was health vulnerability among older adults. Based on a comprehensive literature review, the frailty index (FI) framework was adopted to operationalize this construct. In line with established methodologies, an internationally recognized scoring methodology was applied (23), and indicators were selected according to data availability to develop a measurement system encompassing two dimensions: physical health and psychological health. Specifically, key indicators were selected: body mass index (BMI), physical weakness, self-rated health changes, fall risk, physical discomfort, chronic diseases, emotional characteristics, depression, and anxiety. The overall score for the health vulnerability was computed by assigning values to each indicator: polytomous variables were scored from 1 to 5, and binary variables were assigned values of 0 or 1. For each participant, the scores of all indicators were summed and divided by the theoretical maximum score, which represents the total score achievable if all indicators reached their highest possible value. This calculation yielded a continuous index ranging from 0 to 1, with higher values indicating more severe health vulnerability. This composite metric provided a comprehensive reflection of health vulnerability in the older adult population. Additionally, self-rated health was operationalized as a 5-point ordinal variable (from 1, “very bad,” to 5, “excellent”), allowing direct assessment of individuals’ subjective health perceptions. Other indicators were similarly transformed into categorical variables with defined severity thresholds. This method not only enabled clear differentiation of health states in statistical analyses but also offered dual analytical advantages. On one hand, it visually highlighted disparities across health metrics and identified key determinants of health fragility. On the other hand, its compatibility with standard statistical frameworks significantly improved the interpretability and applicability of the findings.

#### 2.2.2 The independent variable and control variable

The independent variable in this study was access to primary healthcare services, which represents a multidimensional concept. It is defined as the opportunity for, or ease with which, individuals or communities can utilize primary healthcare services in accordance with their needs (24). The evaluation of access typically encompasses geographical, financial, and quality-related dimensions (25). In this questionnaire, access was measured using items such as:” Is it convenient to go to the village clinic (township health centre)?” There were three main options: inconvenient, neutral, and convenient. “Does he/she think the medical fee, inspection fee, pharmaceutical cost at the village clinic (township health centre) are expensive?” etc. The questions are presented in Table 1. These options are assigned scores; a higher score indicates accessibility of primary healthcare services. The control variables were age, gender, education, marriage, income, and living conditions.

**Table 1:** The assessment of variable.

Latent variable	Operating variable	Options and assignment
Physiological vulnerability	Body Mass Index (BMI) A dummy variable indicating whether the respondents are obese	0 = 18.5 ≤ BMI < 24 0.5 = 24 ≤ BMI < 28 1 = BMI < 18.5 or BMI ≥ 28
	Physical discomfort Has he(she) experienced any physical discomfort in the past two weeks?	0 = no 1 = yes
	Physical Weakness Has his(her) health status changed over the past year?	0 = no 1 = yes
	Health changes Has his(her) health status changed over the past year?	0 = no 1 = yes
	Risk of falling Has he(she) fallen down in the past year?	0 = no 1 = yes
	Self-assessed health How is his(her) current self-assessed health status?	1 = very bad 2 = bad 3 = average 4 = good 5 = excellent
	Chronic illnesses Does he(she) have any chronic illnesses?	0 = no 1 = one kind 2 = 2 ~ 3 kinds 3 = 4 kinds and above
Psychological vulnerability	Emotional characteristics Is he(she) able to let go and move on?	0 = yes 1 = no
	Depression and anxiety Is he(she) experiencing feelings of stress, difficulty concentrating, depression, loneliness, etc.?	0 = no 1 = yes
Access to primary healthcare services	Geographical accessibility Is it convenient to go to the village clinic (township health centre)?	1 = inconvenient 2 = neutral 3 = convenient
	Quality Can the village clinic (township health centre) really help with his (her) health issue?	0 = no 1 = yes
	Financial accessibility Does he/she think the medical fee, inspection fee, pharmaceutical cost at the village clinic (township health centre) are expensive?	1 = very expensive 2 = expensive 3 = neutral 4 = reasonably affordable 5 = extremely affordable

### 2.3 Procedure and statistical analysis

This study received ethical review and approval from the Ethics Committee of The First Affiliated Hospital of Air Force Medical University (Xijing Hospital) (approval no: KY20252074-C-1). Prior to survey administration, informed consent was obtained from all participants after a comprehensive explanation of the study objectives. Explicit assurances were provided regarding data confidentiality, and privacy protection measures were implemented in accordance with ethical standards for research. Following data collection, all data were processed and analyzed using SPSS software.

To examine the impact of primary healthcare accessibility on health vulnerability, a structural equation model (SEM) was developed to evaluate its effects on both physiological and psychological vulnerability. In contrast to traditional regression models, SEM allows for the examination of complex causal relationships between latent and observed variables through path analysis. The structural segment of the model depicts the relationships among latent variables, while the measurement component specifies the linkages between latent variables and their manifest indicators. The SEM is formulated as follows:


η=βη+Γξ+ζ

η is the two dependent variables: physiological and psychological vulnerability, ξ is two independent variables. β represents the structural coefficient of the relationship between two latent variables, and Γ represents the structural coefficient of the relationship between latent variables and the explanatory latent variable. ζ represents the prediction error (disturbance term) in the structural model. Meanwhile, we used multi-group SEM to test for heterogeneity. Multi-group SEM can be used to test whether the path coefficients are invariant across different subgroups.

Furthermore, to estimate the loading coefficients between latent and observed variables, we used AMOS 20.0 for constructing and evaluating the SEM, specifically to analyze the influence of primary healthcare accessibility on health vulnerability among older adults.

## 3 Results

### 3.1 Descriptive statistics

Our final sample included a total of 398 older adults. Table 2 shows the characteristics of each participant. The number of male participants was about the same as that of female participants. The majority of participants were married. Specifically, the average age of the respondents was 70.1 years. The percentage of female participants was 54.8%, and that of male participants was 45.2%. There were fewer older people with no formal education, accounting for only 3.0%. Older people were living alone, accounting for 13.1% of the sample. There was no income accounting for 12.3%.

**Table 2:** Participants demographics.

Characteristics	*N*	%
Gender		
Male	180	45.2
Female	218	54.8
Age (years)		
60–69	218	54.8
70–79	137	34.4
≥80	43	10.8
Living condition		
Living alone	52	13.1
Nursing home or care facility	1	0.3
Living with a spouse	216	54.3
Living with relatives	5	1.3
Living with children	124	31.2
Education		
No formal education	12	3.0
Less than primary school	131	32.9
Junior high school	138	34.7
High school and above	117	29.4
Marriage		
Divorced	7	1.8
Bereaved	69	17.3
Single	1	0.3
Married	321	80.7
Income (yuan)		
No income	49	12.3
<1,000	57	14.3
1,000–1999	58	14.6
2000–2,999	82	20.6
3,000–3,999	75	18.8
≥4,000	77	19.3

### 3.2 Health vulnerability

Table 3 summarizes the level of participants’ health vulnerability. On average, the level of physiological vulnerability is above 0.40, presenting a higher score than psychological vulnerability. The highest standard deviation (SD) of psychological vulnerability among the three variables shows the discrepancies in the data. When using the frailty index as an assessment criterion, older adults with scores below 0.25 are classified as having a low level of health frailty, while those with scores equal to or greater than 0.25 are identified as having a high level of health frailty (23). Overall, the level of health vulnerability is above 0.40, suggesting that older adults have some health vulnerability problems to some extent.

**Table 3:** The level of variables.

Variables		Min	Max	Average	SD
Health vulnerability	Physiological vulnerability	0.08	0.83	0.44	0.16
	Psychological vulnerability	0.00	1.00	0.27	0.35
	Health vulnerability	0.07	0.86	0.42	0.16
Access to village clinic	Geographical accessibility	1.00	3.00	2.70	0.58
	Quality	0.00	1.00	0.74	0.44
	Financial accessibility	1.00	5.00	3.59	0.85
Access to township health centre	Geographical accessibility	1.00	3.00	2.64	0.64
	Quality	0.00	1.00	0.84	0.37
	Financial accessibility	1.00	5.00	3.47	0.85

### 3.3 Access to primary healthcare services

Table 3 also reports access to primary healthcare services. The average score of geographical accessibility is above 2.40, the average score for quality is below 0.80, and the average score of financial accessibility is below 4.00. The score indicates that the geographical accessibility is the best among the three measured variables, with an overall level that is higher. Furthermore, access to township health clinics is better than village healthcare centers.

### 3.4 Structural equation modeling

#### 3.4.1 Assessment of the measured models

To examine the effect of access to primary healthcare services on health vulnerability, data standardization was performed to address inconsistencies in responses. The validity of the measurement models for both primary healthcare access and health vulnerability was assessed separately. As summarized in Table 4, reliability and validity tests indicated that all Cronbach’s *α* values exceeded 0.6, demonstrating acceptable reliability. All KMO values were above 0.6, falling within an acceptable range, and Bartlett’s test of Sphericity was significant (*p* < 0.001), supporting the construct validity. Structural equation modeling (SEM) was applied to evaluate both direct and indirect effects among variables (26). In this study, SEM was used to investigate the relationships between accessibility to primary healthcare services and health vulnerability among older rural participants. All indicators in both measurement models met acceptable standards, with most factor loadings reaching statistical significance (*p* < 0.05), providing evidence of construct validity. It should be noted that BMI and health change variables were not significant (*p* > 0.05) and were therefore excluded from the final model. Additionally, composite reliability values also met established standards.

**Table 4:** Reliability and validity test results.

Variables	Cronbach’s *α*	KMO	Bartlett’s test of Sphericity		
			*x* ^2^	*df*	*p*-value
Physiological vulnerability	0.83	0.63	168.25	45	<0.001
Psychological vulnerability	0.60	0.63	1573.13	3	<0.001
Access to village clinic	0.68	0.75	509.04	10	<0.001
Access to township health centre	0.62	0.73	430.35	10	<0.001

#### 3.4.2 Structural equation modeling fitting results

**Figure 1:**
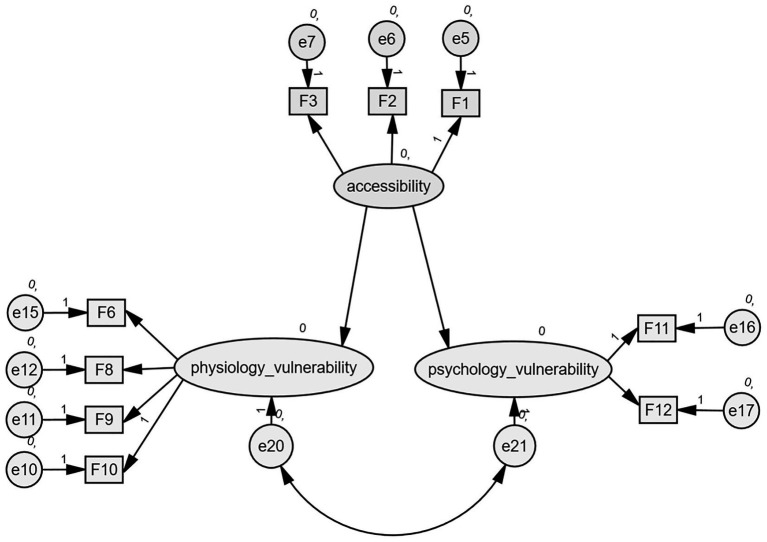
The baseline model.

We evaluated the model fit using Amos 20.0 software. After assigning the indicators to the constructs based on the measurement models, we developed a baseline model, as shown in Figure 1.

The results of the baseline model and multi-group SEM are shown in Table 5. The absolute fit indices were as follows: RMSEA = 0.060, which is less than the threshold of 0.08, and CMIN/DF < 3.000, exceeding the recommended minimum of 0.9, indicating good fit. For the comparative fit indices, the values of IFI, TLI, and CFI all exceed the 0.9 threshold, whereas the value of PNFI is less than 0.5. The effect of access to the village clinic on health vulnerability has been proven, whereas evidence for the township health center remains inconclusive. Overall, the effect on physiological vulnerability is stronger than psychological vulnerability.

**Table 5:** The results of SEM.

Indicator	Baseline model			Multi-group SEM					
	Indicator requirement	Village clinic	Township health centre	Age	Gender	Living condition	Education	Marriage	Income
CMIN/DF	<3.000	1.907	1.280	1.341	1.604	1.589	1.484	1.563	1.232
RMSEA	<0.060	0.048	0.027	0.029	0.039	0.039	0.036	0.038	0.024
*P*-value	<0.050	0.005	0.162	0.056	0.001	0.005	0.002	0.003	0.126
IFI	>0.900	0.950	0.983	0.962	0.901	0.935	0.900	0.938	0.973
TLI	>0.900	0.923	0.974	0.942	0.900	0.902	0.870	0.906	0.958
CFI	>0.900	0.948	0.982	0.960	0.901	0.932	0.895	0.935	0.971
PNFI	>0.500	0.600	0.618	0.590	0.582	0.585	0.550	0.694	0.604

The results of the multi-group SEM show that gender, living conditions, education, and marriage could moderate the effect of access to a village clinic on health vulnerability (*p* < 0.05). First, from the gender dimension, significant heterogeneity is observed between access to primary healthcare services and health vulnerability in both males and females. Through statistical analysis, accessibility to primary healthcare services is shown to have a significant effect on both physiological and psychological vulnerability in women at the 1% significance level (*p* < 0.01). It also exhibits statistically significant associations with males’ physiological vulnerability at the 1% significance level (*p* = 0.002), and with their psychological vulnerability at the 5% significance level (*p* = 0.028).

Second, analysis of living conditions revealed a significant heterogeneity of cohabitation on health vulnerability (*p* < 0.01). However, living alone did not yield statistically significant results in this model, a disparity that may be attributed to the lack of family support and health monitoring among solo-dwelling older adults, which consequently reduces their likelihood of seeking medical care. Moreover, marital status exhibits significant heterogeneity in the relationship between access to primary healthcare services and health vulnerability. However, no significant differences in this heterogeneity were observed between physiological and psychological vulnerability dimensions.

Third, regarding physiological vulnerability, significant associations remain consistent across all educational attainment levels at the 1% significance level (*p* < 0.01). The findings indicate that educational attainment levels exhibit no significant differential effects on physiological vulnerability when accounting for access to primary healthcare services among individuals with varying educational backgrounds. Interestingly, in terms of psychological vulnerability, rural older adults with high school education or above and those with primary school education or below exhibited significant heterogeneity in the relationship between primary healthcare service accessibility and health vulnerability, compared to their counterparts with junior high school education. Notably, these associations were statistically significant at the 5% significance level (Table 6).

**Table 6:** The results of hypothesis testing.

Group name	Variables	Path	Standard regression weight	S. E.	*P*-value
Gender	Male	Accessibility → Physiological Vulnerability	−0.542	0.151	0.002
		Accessibility → Psychological Vulnerability	−0.493	0.191	0.028
	Female	Accessibility → Physiological Vulnerability	−0.711	0.183	***
		Accessibility → Psychological Vulnerability	−0.697	0.185	***
Living condition	Living alone	Accessibility → Physiological Vulnerability	−0.416	0.074	0.305
		Accessibility → Psychological Vulnerability	−0.178	0.096	0.416
	Living with others	Accessibility → Physiological Vulnerability	−0.663	0.173	***
		Accessibility → Psychological Vulnerability	−0.713	0.211	***
Education	Less than primary school	Accessibility → Physiological Vulnerability	−0.577	0.176	0.006
		Accessibility → Psychological Vulnerability	−0.751	0.299	0.010
	Junior High School	Accessibility → Physiological Vulnerability	−0.644	0.214	0.007
		Accessibility → Psychological Vulnerability	−0.656	0.214	0.006
	High school and above	Accessibility → Physiological Vulnerability	−0.755	0.405	0.005
		Accessibility → Psychological Vulnerability	−0.634	0.425	0.021
Marriage	Alone	Accessibility → Physiological Vulnerability	−0.709	0.308	0.007
		Accessibility → Psychological Vulnerability	−0.848	0.341	***
	Married	Accessibility → Physiological Vulnerability	−0.628	0.160	***
		Accessibility → Psychological Vulnerability	−0.714	0.186	***

“***” denote statistical significance at the 1% level.

## 4 Discussion

This study assessed health vulnerability among older adults and found that this population exhibits elevated health risks, with health vulnerability scores substantially exceeding average levels. In contrast, accessibility to primary healthcare services was generally favorable. Using structural equation modeling (SEM), we examined the effect of primary healthcare accessibility on health vulnerability. The results revealed that greater accessibility to primary healthcare services significantly reduced health vulnerability. Notably, accessibility to village clinics demonstrated a stronger effect compared to township health centers. Furthermore, demographic variables, including gender, residence, educational attainment, and marital status, significantly moderated this relationship, indicating substantial heterogeneity in effects across subgroups.

### 4.1 Health vulnerability

The study found a comprehensive health vulnerability index of 0.42 among older adults, with physical and mental health sub-indices measured at 0.44 and 0.27, respectively. All values surpassed the internationally recognized threshold of 0.25 (23), indicating considerable health vulnerability within this population, a result consistent with prior studies (27). Health in older adults can be characterized as a dynamic process of progressive decline associated with aging (28). Common issues such as sedentary behavior, insufficient physical activity, weakened immune function, and a tendency to disregard atypical symptoms or delay medical consultation contribute to the persistence and exacerbation of health vulnerability. As physiological capacity diminishes, older adults frequently experience mental health challenges related to perceived loss and aging, which can provoke negative emotions and intensify psychological vulnerability (9). This psychological vulnerability may, in turn, adversely affect physical health. Older adults, who exhibit high rates of chronic disease and comorbidities (29), often face limitations in activities of daily living due to health impairments. This increases their reliance on family and primary healthcare services, a dependent status that can evoke feelings of guilt or perceived burden, thereby indirectly worsening psychological vulnerability (30). To reduce health vulnerability, several measures should be implemented: actively addressing unhealthy behaviors, maintaining regular physical activity (31), participating in periodic health screenings, and cultivating personal interests and social engagement to strengthen psychological resilience (32). Together, these strategies can alleviate vulnerability across both physical and mental domains, establish a virtuous cycle of health, and ultimately enhance quality of life in later years.

### 4.2 Impact of access to primary healthcare services on health vulnerability

Among the three dimensions of primary healthcare accessibility—geographic, quality-related, and economic—economic accessibility demonstrated the most prominent performance and was consistently maintained at a relatively high level. This can be largely attributed to sustained socioeconomic development and the Chinese government’s vigorous promotion of the basic medical insurance system, which has effectively reduced the financial burden of healthcare on residents (33). Township health centers showed significantly better comprehensive accessibility compared to village clinics, consistent with existing research (34), which may be explained by more equitable resource allocation at the township level (35). Village clinics, which represent the most grassroots yet vulnerable tier of the rural health system, face critical shortages in medical equipment and healthcare personnel. These constraints limit their capacity to meet the diverse, long-term, and comprehensive medical needs of older adults. Moreover, certain medications are not covered by insurance at village clinics, leading many older adults to seek care at township health centers instead. Nevertheless, as the “last kilometer” of healthcare for rural residents, village clinics provide unique advantages in geographic proximity and continuity of patient–provider relationships, enabling more timely and personalized care (36). Thus, they play an indispensable role in enhancing health service accessibility. In the context of China’s health-first strategy and the introduction of multiple supporting policies for primary healthcare development, it is imperative to leverage government initiatives to systematically strengthen village clinics. Efforts should focus on optimizing the allocation of primary medical resources and enhancing the service capacity of primary care institutions. These measures will help solidify the role of village clinics as “health gatekeepers,” reinforce their foundational functions in prevention, diagnosis, treatment, referral, and health education, and tangibly improve the effectiveness of healthcare support for rural residents.

The heterogeneity of demographic variables in the relationship between access to primary healthcare services and vulnerability to health. First, regarding gender differences, the impact of primary healthcare accessibility on health vulnerability was significantly more pronounced among older women than among men. This disparity may be attributed to several factors: women tend to utilize healthcare services more frequently, translate health knowledge into practical behaviors more effectively (37, 38), exhibit greater sensitivity to health risks, and engage more proactively in health self-monitoring (39). They also generally demonstrate higher adherence to medical advice compared to men (40). In contrast, men often pay less attention to health maintenance. Furthermore, older women tend to experience a lower ratio of healthy life expectancy relative to total remaining life expectancy. As a result, healthcare services, which facilitate the transition from unhealthy to healthy states, produce a more substantial marginal effect on health outcomes among older women. As noted by scholar Wei Yan (41), comprehensive medical security and effective disease prevention significantly reduce health poverty vulnerability among rural women, a finding that aligns with and reinforces this study’s result that women benefit more markedly from primary healthcare accessibility. Second, in terms of educational attainment, varying levels of education moderated the effect of primary healthcare accessibility on health vulnerability. Compared to the intermediate education group, both the low and high education groups showed weaker effects on mental health vulnerability. Older adults with low educational levels generally possess a limited capacity to acquire and utilize health information (42). They often seek care at village clinics only for physical symptoms and tend to neglect mental health issues, compounded by generally low health literacy. On the other hand, although highly educated individuals have better health literacy, they often exhibit relatively low trust in primary healthcare services. In addition, the limited capacity of village clinics to provide professional psychological support further attenuates the effect of primary healthcare accessibility on psychological vulnerability in both groups. Third, marital status also significantly moderated the relationship between primary healthcare accessibility and health vulnerability. This may be because married individuals generally benefit from more stable economic conditions, allowing them to seek healthcare without major financial constraints, while a sense of familial responsibility motivates more proactive use of health services (43, 44).

Finally, analysis based on living arrangements indicated that the influence of primary healthcare accessibility on health vulnerability was more pronounced among older adults living with family members, while this association was not statistically significant among those living alone. This finding contrasts with the results reported by Zheng Jiyou et al. in their study on rural empty-nest older adults, which identified healthcare accessibility as a significant factor affecting the health of older adults living alone (45). The discrepancy may be related to methodological differences. The current study relied on proxy questionnaires completed by adult children on behalf of their parents. Although respondents were instructed to accurately report their parents’ health status, children cohabiting with parents likely had more frequent daily interactions and were therefore better informed about their parents’ health conditions. In addition, older adults living with family receive more daily companionship and health-related monitoring, leading to greater health awareness and higher healthcare utilization. Thus, the effect of primary healthcare accessibility appeared more evident in this group. In contrast, older adults living alone often lack regular companionship and health reminders, increasing the likelihood of overlooking mild symptoms or delaying medical consultations. This behavior can allow minor health issues to progress into serious conditions. In such contexts, conventional primary healthcare services may be insufficient to effectively address their needs, which may explain the attenuated role of accessibility in this subgroup. We recognize certain limitations regarding the representation of older adults living alone in this study. Therefore, we emphasize that findings related to this group are preliminary and based on the available sample. Generalizability should be further verified using more diverse research methods in future studies. We avoid over interpreting these results and focus instead on elucidating the relationships between variables. Existing research consistently demonstrates that older adults living alone, often affected by insufficient social support, loneliness, and elevated depression risk (42, 46), exhibit particularly high health vulnerability. The study by Yan Yueping et al. (47) further highlights that depressive mood is the most severe health issue in this population, directly aggravating their mental health vulnerability. Thus, this study suggests that for older adults living alone, social conditions may exert a stronger influence on health vulnerability than primary healthcare accessibility. What is most needed in this group is robust social support and targeted psychological intervention.

In summary, primary healthcare services are well-suited to the health needs of older adults due to their core attributes of accessibility, continuity, and comprehensiveness. As the first line of defense within the healthcare system, primary healthcare plays a vital role in improving health literacy among older adults. It facilitates early detection of health issues and enables timely referrals when necessary, thereby effectively preventing disease progression and reducing the risk of complications (48). For older adults requiring long-term care, primary healthcare institutions can deliver continuous and integrated services by coordinating resources from families, communities, and institutional providers (49). The government should adopt a multidimensional approach to enhance the capacity and accessibility of primary healthcare, with emphasis placed on the following priorities: strengthening resource allocation to village clinics, implementing systematic training programs for village doctors, upgrading age-friendly infrastructure, and expanding health education initiatives at the grassroots level. Particular attention should be directed toward vulnerable groups, including older adults and women (37). Basic medical insurance policies should also be optimized to reduce the financial burden of healthcare on older adults. Through these measures, disparities in primary healthcare accessibility can be diminished, health vulnerability among older adults can be mitigated, and healthy aging can be effectively promoted.

## 5 Limitations

Current research on health vulnerability among older adults remains limited by the multidimensional nature of contributing factors and their complex interactions. Existing vulnerability indicators exhibit shortcomings in capturing dynamic and interactive impact, particularly the underlying link between primary healthcare accessibility and health vulnerability. Furthermore, the use of proxy-completed questionnaires may still raise concerns regarding sample representativeness, particularly among vulnerable groups that are often under-represented in research. This could introduce selection bias, limiting the external validity and generalizability of the study findings. Future studies ought to incorporate metrics such as social support and healthcare accessibility, use longitudinal designs and mixed-methods approaches, and integrate stratified sampling strategies to reduce sample heterogeneity. These refinements would strengthen the external validity and policy relevance of research findings.

## 6 Conclusion

This study found that access to primary healthcare services significantly influences health vulnerability among older adults. Based on a comprehensive field-based assessment, our results indicate that although township health centers demonstrate higher overall accessibility, village clinics play a more essential role due to their greater geographic proximity and the established patient–provider relationships within communities. Therefore, this study recommends actively enhancing the professional capability of village doctors. Furthermore, demographic variables significantly moderate this relationship, indicating that policy formulation should incorporate the diverse needs of different population subgroups. For instance, health education initiatives could be specifically tailored for women; regular home-visit services could be organized for older adults living alone; simplified health education materials could be developed for individuals with lower educational attainment; and digital health guidance could be provided for highly educated groups. Overall, prioritizing resource allocation and service capacity building in village clinics, while emphasizing healthcare equity for vulnerable populations, will significantly contribute to achieving healthy aging goals.

## Data Availability

The original contributions presented in the study are included in the article/supplementary material, further inquiries can be directed to the corresponding authors.
